# Rational Application of Fertilizer Nitrogen to Soil in Combination With Foliar Zn Spraying Improved Zn Nutritional Quality of Wheat Grains

**DOI:** 10.3389/fpls.2018.00677

**Published:** 2018-05-24

**Authors:** Haiyong Xia, Yanfang Xue, Dunyi Liu, Weilin Kong, Yanhui Xue, Yanyan Tang, Jin Li, Dong Li, Peipei Mei

**Affiliations:** ^1^Crop Research Institute, National Engineering Laboratory of Wheat and Maize, Shandong Academy of Agricultural Sciences, Jinan, China; ^2^College of Life Sciences, Shandong Normal University, Jinan, China; ^3^State Key Laboratory of Crop Biology, Shandong Agricultural University, Taian, China; ^4^Maize Research Institute, National Engineering Laboratory of Wheat and Maize, Shandong Academy of Agricultural Sciences, Jinan, China; ^5^College of Resources and Environmental Sciences, Southwest University, Chongqing, China; ^6^College of Life Sciences and Technology, Henan Institute of Science and Technology, Xinxiang, China

**Keywords:** Zinc biofortification, nitrogen fertilizer, foliar application, phytic acid, protein, carbohydrate, calcium, bioavailability

## Abstract

To alleviate human zinc (Zn) deficiency, it is worthy to develop rational agronomic managements to achieve high yielding and high resource-use efficiency wheat (*Triticum aestivum* L.) grains biofortified with Zn. Effects of application of three rates of nitrogen (N) fertilizer (75,200 and 275 kg·ha^−1^) to soil in combination with three foliar applications (deionized water, Zn alone, and a combination of Zn and sucrose) on grain yield, yield components, grain Zn concentration, protein, phytic acid (PA), phosphorus (P), calcium (Ca), and carbon (C), as well as on Zn bioavailability, were investigated in four wheat cultivars (“Jinan 17,” “Jimai 20,” “Jimai 22,” and “Luyuan 502”) under field conditions. Enhanced N increased Zn and protein concentrations as well as bioavailability; excessive N input did not result in further improvements. Zinc spraying was more effective than soil fertilizer N application, the spray of Zn (with or without sucrose) increased grain Zn concentrations by 11.1–15.6 mg·kg^−1^ (27.1–38.1%), and increased grain Zn bioavailability, estimated using total daily absorbed Zn (TAZ) and molar ratios of PA/Zn) and PA × Ca/Zn, by 0.4–0.6 mg d^−1^ (28.6–42.9%), 23.1–27.4% and 24.0–28.0%, respectively. Remarkably, increases caused by ‘Zn + sucrose’ were higher than spraying Zn alone. Grain Zn bioavailability was more sensitive to the selection of cultivar than Zn concentrations. Among cultivars, the higher the grain yields and concentrations of antinutritional compounds, the lower the grain Zn nutritional quality would be. 200 kg N ha^−1^ application rate in combination with foliar spraying of “Zn + sucrose” maximized grain Zn concentrations of “Jinan 17,” “Jimai 20,” “Jimai 22,” and “Luyuan 502” to be 59.4, 56.9, 55.8, and 60.9 mg kg^−1^, respectively, achieving the target value for biofortification. Additionally, PA/Zn and PA × Ca/Zn of “Jinan 17,” “Jimai 20,” and “Luyuan 502” were <15 and 200, and TAZ was maximized to be 2.2, 2.0, and 2.1 mg d^−1^, respectively, indicating higher bioavailability. Therefore, optimal soil N and foliar Zn management together with suitable cultivars maintained high grain yield with lower N input and could substantially increase grain Zn nutritional quality simultaneously.

## Introduction

Zinc (Zn) is an essential micronutrient for the survival of plants, animals and human beings. Zinc deficiency, however, as one of the most widespread nutritional disorders, is prevalent in a lot of regions worldwide, especially in developing countries (Cakmak, 2008). Zinc deficiency causes serious health complications including retarded growth, anorexia, and hypogeusia in children (Brown et al., 2002), as well as pregnancy problems and several chronic diseases in adults (Prasad, 2001; Ota et al., 2012). It has been reported that more than 30% of the whole world's 6 billion people are deficient in Zn (White and Broadley, 2009), and a 1.9% total global burden of disease is caused by Zn deficiency (Rodgers et al., 2004). Annually, around 800,000 people die because of zinc deficiency, of which 450,000 are children under 5 years old (Das and Green, 2013). It has been reported that diarrhea causes 15% of infant deaths, which is dramatically higher than 2% due to HIV/AIDS, number of children below 5 years dying from diarrhea and pneumonia was alarmingly high up to 600,000 in India in 2010, and Zn supplementation could decrease most of children deaths due to diarrhea and pneumonia (United Nations International Children's Emergency Fund, 2012; Das and Green, 2013). In China, dietary deficiency of Zn affects about 100 million people, mostly children below 5 years old and pregnant women living in rural areas (Ma et al., 2005).

Limited food diversity and insufficient dietary Zn intake are responsible for Zn deficiency in human body, especially for those people in developing countries survive and rely on wheat-based diets containing low Zn concentrations (Welch and Graham, 2004). China, as the world's largest consumer and producer of wheat, produced 17.6% of the world's wheat harvest (FAOSTAT, 2016)^1^ Moreover, half Chinese people rely on wheat-dominated foods, especially in northern China. Chen et al. (2017) reported that Zn concentrations in wheat grains ranged between 10.1 and 49.7 mg kg^−1^ with an average of 23.3 mg kg^−1^ in seven major wheat production provinces of China. However, this averaged concentration is much lower than the biofortification target concentration 38 mg kg^−1^ set by the HarvestPlus program (http://www.harvestplus.org; Hao et al., 2015), 40 mg kg^−1^ (Allen et al., 2006; Tang et al., 2008), 45 mg kg^−1^ determined by Ortiz-Monasterio et al. (2007) and Pfeiffer and McClafferty (2007), or 40–50 mg kg^−1^ according to the FAO (Wang et al., 2012). Besides the inherent concentration of Zn being too low to meet daily human requirement, the bioavailability of Zn in wheat grains/flours is also relatively low, because the existing antinutritional phytic acid (PA) and phenolic compounds reduce the biological availability of mineral elements including Zn, iron (Fe), and calcium (Ca) in the human digestive tract (Cakmak et al., 2010b). Additionally, the milling of the wheat grains into white flours (starch-rich endosperm) further reduces the concentration of Zn, because the Zn-enriched outer parts of the grains (mainly the aleurone layer and embryo) have been discarded (Xue et al., 2014a). Therefore, increasing the Zn concentration as well as its bioavailability in wheat grains/flours and promoting wholegrain nutrition is now an urgent need for human health benefits.

Harvesting wheat with a higher concentration of Zn to instill benefits for human health is thought to be obtained more rapidly by the agronomic biofortification approach (e.g., fertilization) than by traditional breeding or genetic engineering methods (Chen et al., 2017). The application of nitrogen (N) fertilizer has been commonly adopted to increase wheat production. Recent studies showed that under both field and glasshouse conditions, no or low N supply led to low wheat grain Zn concentrations, while an optimal N application amount for grain yield caused higher Zn concentrations; however, increasing N supply over the optimal rate did not further increase grain Zn concentrations as well as N concentrations, but caused a decrease in both N and Zn concentrations (Kutman et al., 2010; Shi et al., 2010; Gooding et al., 2012; Xue et al., 2012, 2014b; Chen et al., 2017). It seems that the optimal or adequate N supply significantly enhanced the rate of root Zn uptake and, more evidently, the root-to-shoot transport and re-translocation of radioisotope ^65^Zn from leaves into grains under both controlled and field conditions (Erenoglu et al., 2011). The reason is that N-containing Zn-chelators/transporting proteins may play important roles in Zn uptake, xylem transport, remobilization via phloem, and determining the sink strength for Zn deposition in wheat grains (Uauy et al., 2006; Chen et al., 2017). However, the effect of fertilizer N application to soil in enhancing grain Zn concentration is limited; it plays a more important role in maintaining high grain yield. Compared with soil applications of fertilizer N or Zn, foliar Zn application is much more effective in the enrichment of the whole grain and the endosperm with Zn (Zhang et al., 2010, 2012b; Wang et al., 2012; Zou et al., 2012). Experiments conducted in seven countries (China, India, Kazakhstan, Mexico, Pakistan, Turkey and Zambia) covering 23 sites over 3 years by using 10 different wheat cultivars showed that a 83.5% increase (more than 10 mg·kg^−1^) in grain Zn was achieved by foliar Zn spraying alone, while soil Zn fertilization was less effective (Zou et al., 2012). Zinc concentration in wheat grains was positively correlated with foliar Zn rates (Zhang et al., 2012b), indicating that the grain sink strength and the translocation of Zn to grains are not limiting factors under the given conditions. Can a strategy be implemented to maximize the harvest of grain Zn (concentration and bioavailability) and grain yield at the same time? We speculate that improving grain yield and Zn uptake by sufficient/optimal N application during the vegetative growth stage together with efficient foliar application of Zn to maintain sufficient Zn in wheat shoots for its re-translocation to grains after anthesis under field conditions may maximize grain yields and Zn accumulation in whole grains of wheat. However, such an integrated strategy has not yet been investigated, especially in relation to grain Zn bioavailability and other grain Zn nutritional quality-related traits including concentrations of protein, total and phytate phosphorus (P), and Ca.

The carbohydrate or sucrose status of the plant can influence the transport of Zn into the developing wheat grain. Pearson et al. (1996) observed that the depletion of carbohydrate reserves within cultured ears of wheat (by maintaining them in darkness prior to labeling) reduced the transport of radioisotope ^65^Zn into the grain. Because the grain sink capacity is limited, sucrose at high supply rates may accumulate in the peduncle and chaff, resulting in stomatal closure, the abatement of transportation by the xylem, and finally a decreased accumulation of micronutrients (including Zn) in grains (Ma et al., 1996). Recent studies showed that the grain Zn concentration significantly decreased with increasing sucrose supply to detached ears, due to a dilution effect resulting from the increase in grain weight (Zhang et al., 2012a; Liu et al., 2014). However, it is unknown whether exogenous sucrose supply (with/without Zn) affects the grain Zn concentration, bioavailability, and other Zn-related nutritional traits of wheat without detaching ears grown under real field conditions.

Different wheat cultivars differ in grain yields and nutritional qualities, resulting in different uses. High-quality strong gluten cultivars are suitable for making bread. Flours with mid-level gluten contents are suitable for making dumplings, noodles, or steamed buns. Flours with weak gluten contents are suitable for cookies and pastries. Previous researches have revealed that Zn concentrations in grains of wheat are largely influenced by genotype, environment and their interactions (Gomez-Becerra et al., 2010; Joshi et al., 2010; Murphy et al., 2011). Under Zn-deficient soils, Gomez-Coronado et al. (2016, 2017) found that selecting the high efficient cultivars for Zn accumulation combined with appropriate soil and foliar Zn applications could be a best strategy to biofortify bread wheat with Zn. However, responses in grain Zn concentrations, bioavailability, and other nutritional properties including concentrations of protein, total and phytate P, and Ca of the abovementioned different kinds of wheat cultivars to soil application of fertilizer N in combination with foliar Zn spray have been less studied. Most experiments investigating the relationship between N nutrition and Zn deposition in wheat grains were conducted under soil pot culture or hydroponic conditions, and usually only one cultivar or genotype was used (Gomez-Coronado et al., 2017).

The objectives of this research were (1) to quantify the effects of soil fertilizer N and foliar Zn (with or without sucrose) application on grain yields and yield components; (2) to quantify their efforts on Zn nutritional qualities including Zn concentrations and bioavailability for humans in whole flours; (3) to quantify their effects on protein, total and phytate P, Ca, and carbon (C) concentrations, and on the ratios of C/N and phyate-P/total P in whole flours; and (4) to elucidate relationships among the abovementioned grain Zn-related nutritional traits across different wheat cultivars. The differences in Zn nutritional quality-related parameters among different kinds of wheat genotypes were also evaluated.

## Materials and methods

### Wheat grain biofortification

The field experiment was conducted during the 2013–2014 growing season at Yinmaquan Experimental Station (36.4°N, 117.5°E), Shandong Academy of Agricultural Sciences, China. The area has a typical continental and warm climate, with an annual mean temperature of 13.6°C and a long-term mean annual rainfall of 625 mm. The soil at the site was classified as sandy loam, with a pH of 7.8 (1:2.5 *w/v* in water). The top 20 cm of the soil contained 19 g kg^−1^ organic matter analyzed using the Walkley-Black method (Walkley and Black, 1934). Available N (109 mg kg^−1^), Olsen P (24 mg kg^−1^) and exchangeable K (162 mg kg^−1^) were analyzed by extracting 5.0 g soil with 50 mL 2.0 mol L^−1^ KCl, 100 ml 0.5 mol L^−1^ NaHCO_3_ and 50 mL 1.0 mol L^−1^ NH_4_OAc, respectively (Page et al., 1982). The concentration of diethylenetriaminepentaacetic acid (DTPA)-extractable Zn obtained by extracting 10 g soil (<2 mm) with 20 mL 0.005 mol L^−1^ DTPA + 0.01 mol L^−1^ CaCl_2_ + 0.1 mol L^−1^ TEA (triethanolamine) solution (Lindsay and Norvell, 1978) was 1.5 mg kg^−1^.

The experiment was a split-split-plot design with three factors consisting of three foliar spray treatments (split-split plot), four cultivars (subplot), and three N application rates (main plot) in four replicates. The N application rates were 75, 200, and 275 kg of N ha^−1^, respectively. The four winter wheat (*Triticum aestivum* L.) cultivars were “Jinan 17,” “Jimai 20,” “Jimai 22,” and “Luyuan 502,” respectively. “Jinan 17” is a high-quality strong gluten cultivar, suitable for making bread. “Jimai 20” is suitable for making both bread and noodles. “Jimai 22” is a high-yielding wheat cultivar and is sown over the largest area in contemporary China. “Luyuan 502” is also a high-yielding wheat cultivar, which is widely cultivated in northern China. The three foliar treatments were: (1) foliar spray of deionized water as a control; (2) spray of ZnSO_4_·7H_2_O (0.4%, *w/v*); and (3) a combination spray of ZnSO_4_·7H_2_O (0.4%, *w/v*) and sucrose (3.0%, *w/v*). All solutions, containing 0.01% (*v/v*) Tween 20 as a surfactant, were foliar-applied at 900 L ha^−1^ after sunset. Foliar applications were conducted four times at 5-days intervals, starting from 5 days after wheat flowering. The area of the main plot was 4 × 20 m = 80 m^2^, that of the subplot was 4 × 5 m = 20 m^2^, and that of the split-split plot was 2 × 1 m = 2 m^2^.

A half amount of each N application rate (supplied as urea), and all of the P fertilizer (120 kg of P_2_O_5_ ha^−1^, supplied as calcium superphosphate), K fertilizer (100 kg of K_2_O ha^−1^, supplied as potassium sulfate), and Zn fertilizer (30 kg of ZnSO_4_·7H_2_O ha^−1^) were evenly distributed and incorporated into the upper 20 cm of the soil prior to wheat planting. The remaining half of the N was top-dressed with irrigation at the jointing stage. All plots were adequately irrigated at stages of pre-wintering, stem elongation and flowering, and weeded manually. There were no fungicides applied during the growth period. At the booting stage, omethoate (2-dimethoxyphosphinoylthio-N-methylacetamide) (Dazhou Xinglong Chemical Co., Ltd., Dazhou, China) was sprayed to control aphids.

At maturity, a 1-m^2^ area of wheat spikes aboveground in the center of each split-split plot subjected to foliar spraying treatments was removed by hand for determination of grain yield and yield components. The number of spikes in the 1 m^2^ area was counted; 30 random spikes were used to count and calculate the averaged kernel number per spike. All grains were manually separated from the husks, oven-dried at 70°C and then weighed to determine the 1,000 kernel weight and grain yield.

### Nutrient analysis

After being washed quickly with deionized water, about 1 kg of grain samples were dried in an oven at 60–65°C for 72 h and then ground with a stainless-steel grinder (RT-02B, Chinese Taipei). Ground samples were digested with HNO_3_-H_2_O_2_ in a closed microwave digester (CEM, Matthews, North Carolina, USA). The concentrations of nutrients (P, Zn, and Ca) in the digests were determined by inductively coupled plasma atomic emission spectroscopy (ICP-AES, OPTIMA 3300 DV, Perkin-Elmer, Waltham, Massachusetts, USA). A reference grain sample IPE556 from Wageningen University was included in each batch to ensure analytical quality. Phytate-P concentration was analyzed according to the method of Haug and Lantzsch (1983). The carbon concentration and ratio of C/N was determined using Multi N/C 3100 (Analytik Jena AG, Germany). Grain protein concentration on a dry weight basis was determined by a near infrared transmittance analyzer Foss-Tecator 1241 (Foss, Hoganas, Sweden), which was calibrated by the Kjeldahl method according to Approved Method 46–12 (American Association for Clinical Chemistry, 2000).

### Estimation of Zn bioavailability

The molar ratio of PA/Zn has been widely used as a simplified indicator of Zn bioavailability in the human diet (Morris and Ellis, 1982; Ryan et al., 2008; Liu et al., 2017). Calcium may accentuate the effect of PA on Zn bioavailability. As a result, the molar ratio of PA × Ca/Zn has also been adopted as a measure of Zn bioavailability (Ellis et al., 1987). Phytate-P was converted to PA by dividing by 0.282 to calculate the molar ratios of PA/Zn and PA × Ca/Zn. A molar ratio of PA/Zn below the critical ratio of 15 represented about 35% Zn availability (World Health Organization, 1996). A molar ratio of PA × Ca/Zn below the critical ratio of 200 indicated a good Zn bioavailability (Ellis et al., 1987).

The human body regulates Zn homeostasis through gastrointestinal secretion and excretion of endogenous Zn in addition to absorption of exogenous Zn (Lim et al., 2013). A trivariate model based on Zn homeostasis in the human intestine has also been widely adopted to evaluate Zn bioavailability (Miller et al., 2007; Liu et al., 2017):

$$\begin{array}{l} TAZ=0.5\;\times \;65\;\times \;100\;\times \;\left\{A_{MAX}+TDZ+K_{R}\;\times \;\left(1+\frac{TDP}{K_{P}}\right)-\sqrt{{\left(A_{MAX}+TDZ+K_{R}\times \left(1+\frac{TDP}{K_{P}}\right)\;\right)}^{2}-4\;\times \;A_{MAX}\;\times \;TDZ\;}\right\} \end{array}$$

where *TAZ* (total daily absorbed Zn, mg Zn d^−1^) was based on reference adults consuming wheat flour (0.3 kg d^−1^) as a sole daily source of Zn and phytate (Rosado et al., 2009); *AMAX* = maximum Zn absorption; *TDZ* (total daily dietary Zn, mmol Zn d^−1^) = Zn concentration in wheat flour (mg kg^−1^) × reference adults consuming wheat flour (0.3 kg d^−1^) ÷ the relative atomic mass of Zn (65 g mol^−1^); *K*_*R*_ = equilibrium dissociation constant of the Zn-receptor binding reaction; *TDP* (total daily dietary PA, mmol PA Ld^−1^) = PA concentration in wheat flour (mg kg^−1^) × reference adults consuming wheat flour (0.3 kg d^−1^) ÷ the relative molecular mass (660.04 g mol^−1^); and K_P_ = equilibrium dissociation constant of the Zn–PA binding reaction (Liu et al., 2017). The three parameters regarding Zn homeostasis in the human intestine, AMAX, KR, and KP, have their constant values of 0.091, 0.680, and 0.033, respectively (Hambidge et al., 2010).

### Statistical analysis

Data were subjected to ANOVA using SAS software (SAS 8.0, SAS Institute, Cary, North Carolina, USA) and means were compared by Fisher's protected least significant difference (LSD) at *P* ≤ 0.05, 0.01, or 0.001. SPSS software (17.0) was used for calculating Pearson correlation coefficients.

## Results

### Grain yields and yield components

Increasing the N application rate from 75 to 200 kg ha^−1^ and from 200 to 275 kg ha^−1^, decreased grain yields gradually from the initial 7719.7 kg ha^−1^ to the final 7226.6 kg ha^−1^, and decreased spike numbers from 449.1 to 415.5 10^4^ ha ^−1^ (Table 1). Compared to the low N supply (75 kg N ha^−1^), the 1,000 kernel weight was significantly increased to its maximum value of 45.6 g by 200 kg N ha^−1^. No significant effects of N on the kernel number per spike were observed. Among different wheat cultivars, grain yields varied from 7073.3 to 7703.0 kg ha^−1^. ‘Luyuan’ 502 had the lowest grain yield, which was significantly lower than that of “Jimai 20,” and “Jimai 22” had the maximum yield. Spike numbers are in the order of “Jinan 17” > “Jimai 20” > “Jimai 22” > “Luyuan 502,” and varied dramatically from 503.2 to 345.2 10^4^ ha^−1^, while the kernel per spike and the 1,000 grain weight were the reverse of the previous order, varying from 32.5 to 40.2 g and from 41.5 to 48.9 g, respectively. As expected, foliar treatments had non-significant impacts on grain yields and yield components (Table 1).

**Table 1:** Effects of nitrogen and foliar applications on grain yields and yield components of different wheat cultivars.

**Treatments**	**Grain yield (kg ha^−1^)**	**Spike number (10^4^ ha^−1^)**	**Kernel number per spike**	**1,000 kernel weight (g)**
**N APPLICATION RATE (N)**				
75 kg ha^−1^	7719.7a	449.1a	36.5a	44.7b
200 kg ha^−1^	7419.1ab	424.9b	35.1a	45.6a
275 kg ha^−1^	7226.6b	415.5b	34.2a	45.0ab
LSD_0.05_	393.2	21.7	3.3	0.9
**CULTIVAR (C)**				
Jinan 17	7417.1ab	503.2a	32.5b	41.5d
Jimai 20	7627.3a	460.5b	33.2b	44.3c
Jimai 22	7703.0a	410.6c	35.1b	45.7b
Luyuan 502	7073.3b	345.2d	40.2a	48.9a
LSD_0.05_	453.9	25.1	3.8	1.0
**FOLIAR APPLICATION (F)**				
Deionized water	7460.7a	432.8a	34.3a	45.4a
ZnSO_4_·7H_2_O	7385.5a	426.7a	35.1a	44.7a
ZnSO_4_·7H_2_O + Sucrose	7519.3a	430.1a	36.4a	45.2a
LSD_0.05_	393.2	21.7	3.3	0.9
**ANOVA**				
N	0.0473	0.0083	0.3855	0.1103
C	0.0326	<0.0001	0.0004	<0.0001
F	0.7958	0.8521	0.4339	0.3554
N × C	0.4600	0.1859	0.5267	0.1208
N × F	0.4483	0.6647	0.5260	0.4414
C × F	0.9760	0.9484	0.3890	0.4880
N × C × F	0.7522	0.9772	0.6243	0.4753

*Values followed by different lowercase letters in the same column are significantly different among treatments at P ≤ 0.05. Values under ANOVA are probabilities (P values) of the source of variation*.

### Zn concentrations, Zn yields, and estimated Zn bioavailability in whole flours

Adequate N application (200 kg N ha^−1^) significantly increased grain Zn concentration by 9.9%, from 47.5 to 52.2 mg kg^−1^. It also increased Zn yield and Zn bioavailability as compared with low N application (75 kg N ha^−1^), estimated by TAZ, PA/Zn, and PA × Ca/Zn (Table 2). The molar ratio of PA × Ca/Zn was reduced to be <200, indicating a good Zn bioavailability. When compared with adequate N treatment, high N treatment (275 kg N ha^−1^) led to slightly lower grain Zn concentration and yield, and did not make an extra contribution to grain Zn bioavailability.

**Table 2:** Effects of nitrogen and foliar applications on Zn concentrations, Zn yields, total daily absorbed Zn (TAZ), and molar ratios of PA/Zn and PA × Ca/Zn in grains of different wheat cultivars.

**Treatments**	**Zn concentrations (mg kg^−1^)**	**Zn yields (g ha^−1^)**	**TAZ (mg d^−1^)**	**PA/Zn**	**PA × Ca/Zn**
**N APPLICATION RATE (N)**					
75 kg ha^−1^	47.5c	367.8ab	1.6b	18.5a	203.4a
200 kg ha^−1^	52.2a	383.9a	1.8a	16.7b	188.3b
275 kg ha^−1^	49.6b	357.6b	1.8a	16.6b	189.3b
LSD_0.05_	1.6	19.0	0.1	0.8	11.4
**CULTIVAR (C)**					
Jinan 17	50.4b	374.1a	2.0a	14.7c	140.1c
Jimai 20	47.0c	358.5a	1.7b	17.0b	194.5b
Jimai 22	48.9b	376.4a	1.4c	20.7a	249.1a
Luyuan 502	52.9a	370.1a	1.8b	16.7b	190.9b
LSD_0.05_	1.8	22.0	0.1	1.0	13.1
**FOLIAR APPLICATION (F)**					
Deionized water	40.9c	303.7c	1.4c	20.8a	234.2a
ZnSO_4_·7H_2_O	52.0b	382.0b	1.8b	16.0b	178.1b
ZnSO_4_·7H_2_O + Sucrose	56.5a	423.7a	2.0a	15.1c	168.6b
LSD_0.05_	1.6	19.0	0.1	0.8	11.4
**ANOVA**					
N	<0.0001	0.0251	0.0018	<.0001	0.0153
C	<0.0001	0.3819	<0.0001	<0.0001	<0.0001
F	<0.0001	<0.0001	<0.0001	<0.0001	<0.0001
N × C	0.0628	0.0315	0.9132	0.3785	0.1442
N × F	0.7696	0.1596	0.2093	0.0067	0.1459
C × F	0.6965	0.7337	0.0109	0.0002	<0.0001
N × C × F	0.7136	0.9196	0.4234	0.5387	0.7733

*Values followed by different lowercase letters in the same column are significantly different among treatments at P ≤ 0.05. Values under ANOVA are probabilities (P values) of the source of variation*.

Wheat cultivars greatly differed in grain Zn concentration (47.0–52.9 mg kg^−1^), TAZ (1.4–2.0 mg d^−1^), and molar ratios of PA/Zn (15.1–20.8) and PA × Ca/Zn (168.6–234.2) (Table 2). “Jimai 20” had the lowest grain Zn concentration, “Luyuan 502” had the highest. “Jinan 17,” as a high-quality strong gluten wheat cultivar, had the highest TAZ and lowest molar ratios of PA/Zn and PA × Ca/Zn; in contrast, the high-yielding “Jimai 22” sown over the largest area in contemporary China had the lowest TAZ and highest molar raios of PA/Zn and PA × Ca/Zn. There were no significant differences in grain Zn yields among different wheat cultivars under the given conditions.

For foliar Zn-biofortified treatments, the grain Zn concentration and TAZ were significantly increased from the initial 40.9 to 52.0 mg kg^−1^ and from the initial 1.4 to 1.8 mg d^−1^, respectively, by Zn-only treatment, and significantly increased further from 52.0 to 56.5 mg kg^−1^ and from 1.8 to 2.0 mg d^−1^, respectively, by “Zn + sucrose” treatment (Table 2). Similar results were found in grain Zn yields. In contrast, Zn-only treatment and “Zn + sucrose” decreased the molar ratios of PA/Zn by 23.1 and 27.4%, respectively, and decreased the molar ratios of PA × Ca/Zn by 24.0 and 28.0%, respectively.

In addition, as ANOVA indicates, there are some significant interactions between treatments of N applications and cultivars, between N and foliar applications, and between cultivars and foliar applications (Tables 2, 3). N applications × cultivars interaction significantly affected grain Zn yields. At all foliar treatments, no significant differences in grain Zn yields were found among different wheat cultivars at low and adequate N supply, but at high N supply, “Jimai 20” had the lowest grain Zn yield, which was significant lower than that of “Luyuan 502” treated by foliar spraying of deionized water and that of “Jimai 22” treated by foliar Zn-only spraying (Table 3). Effects of foliar applications on molar ratios of PA/Zn differed at different N supply rates. Significant interaction was found between N and foliar applications. For example, at low N supply, a mix of Zn and sucrose resulted in significant decreases in molar ratios of PA/Zn of “Jinan 17” and “Luyuan 502,” as compared with the spray of Zn-only treatment, but at adequate and high N supply, there were no significant differences (Table 3). For cultivars × foliar applications interaction, the effect turned out to be significant on all grain Zn bioavailability traits including TAZ, and molar ratios of PA/Zn and PA × Ca/Zn. As compared with the spray of deionized water, foliar spraying of Zn-only significantly increased TAZ of “Luyuan 502” at low N supply, foliar spraying of Zn and sucrose together led to a further significant increase as compared with the Zn-only treatment (Table 3). Simultaneously, molar ratios of PA/Zn and PA × Ca/Zn of “Luyuan 502” treated by foliar spraying of Zn-only were significantly lower than those treated by deionized water at low N supply, and were further significantly reduced by a combination of “Zn + sucrose” spraying. However, different wheat cultivars showed inconsistent results in terms of TAZ, and molar ratios of PA/Zn and PA × Ca/Zn as affected by foliar applications across all N treatments.

**Table 3:** Effects of nitrogen and foliar applications on Zn concentrations, Zn yields, total daily absorbed Zn (TAZ), and molar ratios of PA/Zn and PA × Ca/Zn in grains of different wheat cultivars.

**N application rate (kg ha^−1^)**	**Cultivar**	**Zn concentrations (mg kg^−1^)**			**Zn yields (g ha^−1^)**			**TAZ (mg d^−1^)**			**PA/Zn**			**PA** × **Ca/Zn**		
		**DW^a^**	**Zn^b^**	**Zn + S^c^**	**DW**	**Zn**	**Zn + S**	**DW**	**Zn**	**Zn + S**	**DW**	**Zn**	**Zn + S**	**DW**	**Zn**	**Zn + S**
75	Jinan 17	39.3DEFc	49.2CDb	56.7ABCDa	289.0ABCc	384.0ABCb	441.9Aa	1.6BCDb	1.8ABCb	2.3ABa	18.2DEFa	15.7BCa	12.4Cb	173.5FGa	153.3CDab	109.7Db
	Jimai 20	38.7EFGb	49.5CDa	52.4BCDa	263.9BCb	423.8Aa	440.8Aa	1.2EFb	1.7BCDa	1.9BCDa	23.8Ba	17.0ABb	15.4Bb	270.5ABCa	202.8ABb	175.9Bb
	Jimai 22	35.7FGb	48.3Da	52.2CDa	288.5ABCb	370.4ABCa	418.5ABa	1.0Fb	1.5Da	1.5Da	27.7Aa	19.0Ab	19.6Ab	303.1Aba	215.9Ab	228.4Ab
	Luyuan 502	38.6EFGc	51.2BCDb	58.7ABCa	285.6ABCc	368.7ABCb	438.1Aa	1.3DEFc	1.6CDb	2.2ABa	22.5BCa	18.1Ab	13.1BCc	255.2BCDa	201.1ABb	151.2BCc
200	Jinan 17	43.3BCDc	54.6Bb	59.4ABCa	322.9ABCb	399.3Aba	453.3Aa	1.9Ab	2.1Aab	2.2ABa	15.3Fa	13.7Cab	12.9BCb	147.5Ga	128.9Dab	121.6Db
	Jimai 20	40.3CDEc	50.8BCDb	56.9ABCDa	336.1Ab	381.4ABCab	431.8Aa	1.5BCDEb	1.7BCDab	2.0ABCa	19.1CDEa	16.6ABab	14.2BCb	224.4CDEFa	195.5ABa	157.2Bb
	Jimai 22	43.8ABCb	53.3BCa	55.8ABCDa	332.7ABb	405.4Aba	447.2Aa	1.2EFb	1.7BCDa	1.5Dab	23.9Ba	17.3ABb	19.9Ab	305.8ABa	202.4ABc	235.6Ab
	Luyuan 502	47.7Ab	60.0Aa	60.9Aa	317.7ABCb	380.8ABCa	398.4ABa	1.5BCDEb	2.1Aa	2.0ABCa	19.4CDEa	13.7Cb	14.0BCb	216.1DEFa	158.0CDb	166.7Bb
275	Jinan 17	40.0CDEFb	51.8BCDa	59.7ABa	296.4ABCb	352.9BCab	426.8ABa	1.7ABa	1.9ABa	2.5Aa	16.7EFa	15.0BCa	12.3Ca	156.9Ga	145.6CDa	123.9CDa
	Jimai 20	34.9Gb	48.5Da	50.5Da	257.1Cb	340.4Ca	351.0Ba	1.6BCc	1.9ABb	2.1ABa	18.1DEFa	15.2BCb	13.3BCb	199.8EFGa	171.0BCab	153.4Bb
	Jimai 22	42.9BCDEc	51.7BCDb	56.3ABCDa	312.2ABCb	400.7ABa	412.1ABa	1.2EFb	1.7BCDa	1.6CDa	24.1ABa	16.6ABb	18.2Ab	310.2Aa	204.8ABb	235.8Ab
	Luyuan 502	45.8ABb	54.6Ba	58.4ABCa	342.0Ab	375.8ABCab	424.1ABa	1.4CDEb	2.1Aa	1.9BCDa	20.8BCDa	13.6Cb	15.3Bb	247.8CDEa	157.6CDb	164.2Bb

a *Deionized water (DW)*.

b *ZnSO_4_·7H_2_O (Zn)*.

c *Sucrose (S). Values followed by different capital letters in the same column are significantly different among treatments at P ≤ 0.05; Values followed by different lowercase letters in the same row for each parameter are significantly different among treatments at P ≤ 0.05*.

On average, the N application rate of 200 kg ha^−1^ was adequate to maintain high grain Zn concentrations, Zn yields and Zn bioavailability (Tables 2, 3). At the 200 kg N ha^−1^ application rate combined with foliar spray of “Zn + sucrose,” grain Zn concentrations of “Jinan 17,” “Jimai 20,” “Jimai 22,” and “Luyuan 502” were maximized to be 59.4, 56.9, 55.8, and 60.9 mg kg^−1^, respectively, achieving the biofortification target value of 38–50 mg kg^−1^ (Table 3). In addition, the molar ratios of PA/Zn and PA × Ca/Zn of “Jinan 17,” “Jimai 20,” and “Luyuan 502” were lower than the critical values of 15 and 200, respectively, suggesting higher Zn bioavailability (Table 3).

### Concentrations of protein, total and phytate phosphorus, calcium, and other Zn-related nutritional traits in whole flours

Compared to the control treatment of low N supply, adequate N treatment significantly increased the protein concentration from 13.3 to 13.7%, and significantly decreased the ratio of C/N from 18.2 to 17.6 (Table 4). When compared with adequate N application, high N application did not make an extra contribution to the protein concentration or the ratio of C/N. However, excessive N application led to a significant lower phytate-P concentration than the adequate N supply. Variation in N supply had non-significant impacts on grain concentrations of C, total P, and Ca, and on the ratio of phytate-P/total P.

**Table 4:** Effects of nitrogen and foliar applications on grain Zn nutritional quality related traits of different wheat cultivars.

**Treatments**	**Protein concentration (%)**	**Carbon concentration (%)**	**C/N**	**Phytate-P concentration (g kg^−1^)**	**Total P concentration (g kg^−1^)**	**Phytate-P/total P (%)**	**Ca concentration (mg kg^−1^)**
**N APPLICATION RATE (N)**							
75 kg ha^−1^	13.3b	42.7a	18.2a	2.44ab	3.64a	67.9a	436.5a
200 kg ha^−1^	13.7a	42.8a	17.6b	2.45a	3.86a	64.3a	447.2a
275 kg ha^−1^	13.7a	42.8a	17.7b	2.32b	3.63a	65.3a	449.7a
LSD_0.05_	0.2	0.2	0.2	0.12	0.24	4.61	14.1
**CULTIVAR (C)**							
Jinan 17	13.5b	43.0a	18.0a	2.07d	3.33b	63.5b	379.9c
Jimai 20	13.7ab	42.7b	17.6b	2.23c	3.51b	64.9b	458.7b
Jimai 22	13.8a	42.7b	17.5b	2.84a	4.01a	71.5a	482.5a
Luyuan 502	13.2c	42.7b	18.2a	2.46b	4.00a	63.3b	456.6b
LSD_0.05_	0.2	0.2	0.2	0.14	0.28	5.32	16.2
**FOLIAR APPLICATION (F)**							
Deionized water	13.5b	42.8ab	17.9a	2.43a	3.91a	63.3a	445.0a
ZnSO_4_·7H_2_O	13.5b	42.9a	17.8ab	2.37a	3.61b	66.7a	444.8a
ZnSO_4_·7H_2_O + Sucrose	13.7a	42.7b	17.7b	2.41a	3.61b	67.5a	443.6a
LSD_0.05_	0.2	0.2	0.2	0.12	0.24	4.61	14.1
**ANOVA**							
N	<0.0001	0.3318	<0.0001	0.0740	0.1117	0.2940	0.1471
C	<0.0001	0.0464	<0.0001	<0.0001	<0.0001	0.0077	<0.0001
F	0.0152	0.1116	0.0616	0.5777	0.0251	0.1656	0.9791
N × C	0.4035	0.5817	0.0212	0.0388	0.7093	0.2701	0.0726
N × F	0.7960	0.5743	0.9620	0.3695	0.1006	0.1008	0.8347
C × F	0.4111	0.9688	0.9613	0.0019	0.2355	0.4148	0.8327
N × C × F	0.2448	0.2470	0.9187	0.3903	0.8891	0.6440	0.8327

*Values followed by different lowercase letters in the same column are significantly different among treatments at P ≤ 0.05. Values under ANOVA are probabilities (P values) of the source of variation*.

Analysis of variance revealed significant effects of wheat cultivars on grain concentrations of protein (13.2–13.8%), C (42.7–43.0%), phytate-P (2.07–2.84 g kg^−1^), total P (3.33–4.01 g kg^−1^), and Ca (379.9–482.5 mg kg^−1^), as well as on the ratios of C/N (17.5–18.2) and phytate-P/total P (63.3–71.5%). “Luyuan 502” had a significantly lower protein concentration than the other three wheat cultivars; in addition, the protein concentration of “Jinan 17” was significantly lower than “Jimai 22.” “Jinan 17” had relatively higher C concentration than “Jimai 20,” “Jimai 22,” and “Luyuan 502.” The ratios of C/N of “Jinan 17” and “Luyuan 502” were significantly higher than those of “Jimai 20” and “Jimai 22.” “Jinan 17,” as a high-quality strong gluten wheat cultivar, had the lowest concentrations of phytate-P, total P, and Ca; in contrast, the high-yielding “Jimai 22” had the highest concentrations of phytate-P, total P, and Ca, and the highest ratio of phytate-P/total P (Table 4).

Compared with the control, foliar Zn or “Zn + sucrose” application had no effects on whole flour traits including concentrations of phytate-P and Ca, and the ratio of phytate-P/total P, but significantly decreased total P concentrations and gradually decreased the ratio of C/N in whole flours. “Zn + sucrose” significantly increased the protein concentration as compared to the control and Zn-only treatment, but significantly reduced the carbon content as compared to the Zn-only treatment (Table 4).

The interaction of fertilizer N applications × cultivars significantly affected C/N ratios (Tables 4, 5). C/N ratios of “Luyuan 502” were significantly higher than those of “Jimai 20” at low N supply, but not at adequate and high N supply across all foliar treatments; C/N ratios of “Luyuan 502” were significantly higher than those of “Jimai 22” at low and high N supply, but not at the adequate N supply (Table 5). N applications × cultivars interaction as well as cultivars × foliar applications interaction significantly affected phytate-P concentrations (Tables 4, 5). For example, the phytate-P concentration of “Luyuan 502” treated by foliar application of “Zn + sucrose” was significantly higher than that of “Jinan 17” or “Jimai 20” at the high N supply, but not at low and adequate N supply (Table 5). Different cultivars had different responses to foliar treatments. The spray of Zn-only and “Zn + sucrose” significantly increased phytate-P concentrations of “Jinan 17” as compared to the control treatment of deionized water, whereas other cultivars showed non-significant differences at the adequate N supply.

**Table 5:** Effects of nitrogen and foliar applications on C/N ratios and phytate-P concentrations in grains of different wheat cultivars.

**N application rate (kg ha^−1^)**	**Cultivar**	**C/N**			**Phytate-P concentration (g kg^−1^)**		
		**DW^a^**	**Zn^b^**	**Zn + S^c^**	**DW**	**Zn**	**Zn + S**
75	Jinan 17	18.1Ba	18.3ABa	18.0ABa	2.04DEa	2.21BCa	1.99Ea
	Jimai 20	18.0BCa	17.5CDa	17.8BCDa	2.63ABCa	2.41ABCa	2.30CDEa
	Jimai 22	18.0BCa	17.9BCa	17.7BCDa	2.83ABa	2.62ABa	2.93ABa
	Luyuan 502	18.9Aab	19.0Aa	18.6Ab	2.48BCDab	2.64ABa	2.20CDEb
200	Jinan 17	18.1Ba	17.8BCDb	17.8BCDb	1.89Eb	2.14Ca	2.19CDEa
	Jimai 20	17.7BCa	17.6BCDa	17.3DEa	2.21CDEa	2.41ABCa	2.30CDEa
	Jimai 22	17.3Ca	17.4CDa	17.4CDEa	2.99Aa	2.65Aa	3.13Aa
	Luyuan 502	17.9BCa	17.5CDa	17.7BCDa	2.63ABCa	2.38ABCa	2.44CDa
275	Jinan 17	18.1Ba	18.1BCa	17.7BCDb	1.91Ea	2.24ABCa	2.04DEa
	Jimai 20	17.8BCa	17.6CDa	17.5BCDEa	1.81Eb	2.11Ca	1.93Eab
	Jimai 22	17.2Ca	17.2Da	17.1Ea	2.96Aa	2.46ABCa	2.95ABa
	Luyuan 502	18.3ABa	18.0BCa	17.9BCa	2.74ABa	2.12Ca	2.55BCa

a *Deionized water (DW)*.

b *ZnSO_4_·7H_2_O (Zn)*.

c *Sucrose (S). Values followed by different capital letters in the same column are significantly different among treatments at P ≤ 0.05; values followed by different lowercase letters in the same row for each parameter are significantly different among treatments at P ≤ 0.05*.

### Relationships among grain Zn nutritional quality-related parameters

Grain Zn concentrations were positively correlated with parameters of TAZ, 1,000 kernel weights, Zn yields, and protein concentrations, but negatively correlated with spike numbers, grain yields, C/N ratios, and molar ratios of PA/Zn and PA × Ca/Zn under field conditions (Table 6). Total daily absorbed Zn was also positively correlated with Zn yields, but negatively correlated with 1000 kernel weights, phytate-P, total P, phytate-P/total P, Ca concentration, and molar ratios of PA/Zn and PA × Ca/Zn.

**Table 6:** Pearson correlation coefficients among grain zinc concentration (ZnC), total daily absorbed Zn (TAZ), spike number (SN), kernel number per spike (KNPS), 1,000 kernel weight (1,000 KW), grain yield (Y), Zn yield (ZnY), carbon concentration (CC), protein concentration (PC), C/N, phytate-P concentration (PA-P), total P concentration (TP), PA-P/TP, calcium concentration (CaC), and molar ratios of PA/Zn and PA × Ca/Zn in different wheat genotypes (*n* = 144).

**Parameters**	**TAZ**	**SN**	**KNPS**	**1000 KW**	**Y**	**ZnY**	**CC**	**PC**	**C/N**	**PA-P**	**TP**	**PA-P/TP**	**CaC**	**PA/Zn**	**PA × Ca/Zn**
ZnC	0.633^***^	−0.249^**^	ns	0.171^*^	−0.179^*^	0.729^***^	ns	0.242^**^	−0.315^***^	ns	ns	ns	ns	−0.634^***^	−0.478^***^
TAZ	-	ns	ns	−0.231^**^	ns	0.498^***^	ns	ns	ns	−0.680^***^	−0.247^**^	−0.415^***^	−0.351^***^	−0.929^***^	−0.867^***^
SN	-	-	−0.233^**^	−0.628^***^	0.579^***^	0.213^**^	0.177^*^	ns	0.212^*^	−0.339^***^	−0.318^***^	ns	−0.386^***^	ns	−0.207^*^
KNPS	-	-	-	0.266^***^	ns	ns	ns	−0.212^*^	ns	ns	ns	ns	0.166^*^	ns	ns
1,000 KW	-	-	-	-	ns	ns	−0.199^*^	ns	ns	0.443^***^	0.367^***^	ns	0.454^***^	0.216^**^	0.339^***^
Y	-	-	-	-	-	0.530^***^	ns	ns	0.237^**^	ns	ns	ns	ns	ns	ns
ZnY	-	-	-	-	-	-	ns	ns	ns	ns	ns	ns	ns	−0.508^***^	−0.378^***^
CC	-	-	-	-	-	-	-	ns	ns	−0.193^*^	ns	ns	ns	ns	ns
PC	-	-	-	-	-	-	-	-	−0.621^***^	ns	ns	ns	ns	ns	ns
C/N	-	-	-	-	-	-	-	-	-	−0.269^***^	ns	ns	−0.303^***^	ns	ns
PA-P	-	-	-	-	-	-	-	-	-	-	0.372^***^	0.599^***^	0.513^***^	0.694^***^	0.762^***^
TP	-	-	-	-	-	-	-	-	-	-	-	−0.511^***^	0.297^***^	0.303^***^	0.360^***^
PA-P/TP	-	-	-	-	-	-	-	-	-	-	-	-	0.210^*^	0.379^***^	0.389^***^
CaC	-	-	-	-	-	-	-	-	-	-	-	-	-	0.327^***^	0.636^***^
PA/Zn	-	-	-	-	-	-	-	-	-	-	-	-	-	-	0.932^***^
PA × Ca/Zn	-	-	-	-	-	-	-	-	-	-	-	-	-	-	-

*“-” indicates no this value*,

“*”, “**”, and “***” *indicate significant correlations at P ≤ 0.05, P ≤ 0.01, and P ≤ 0.001, respectively*.

**Figure 1:**
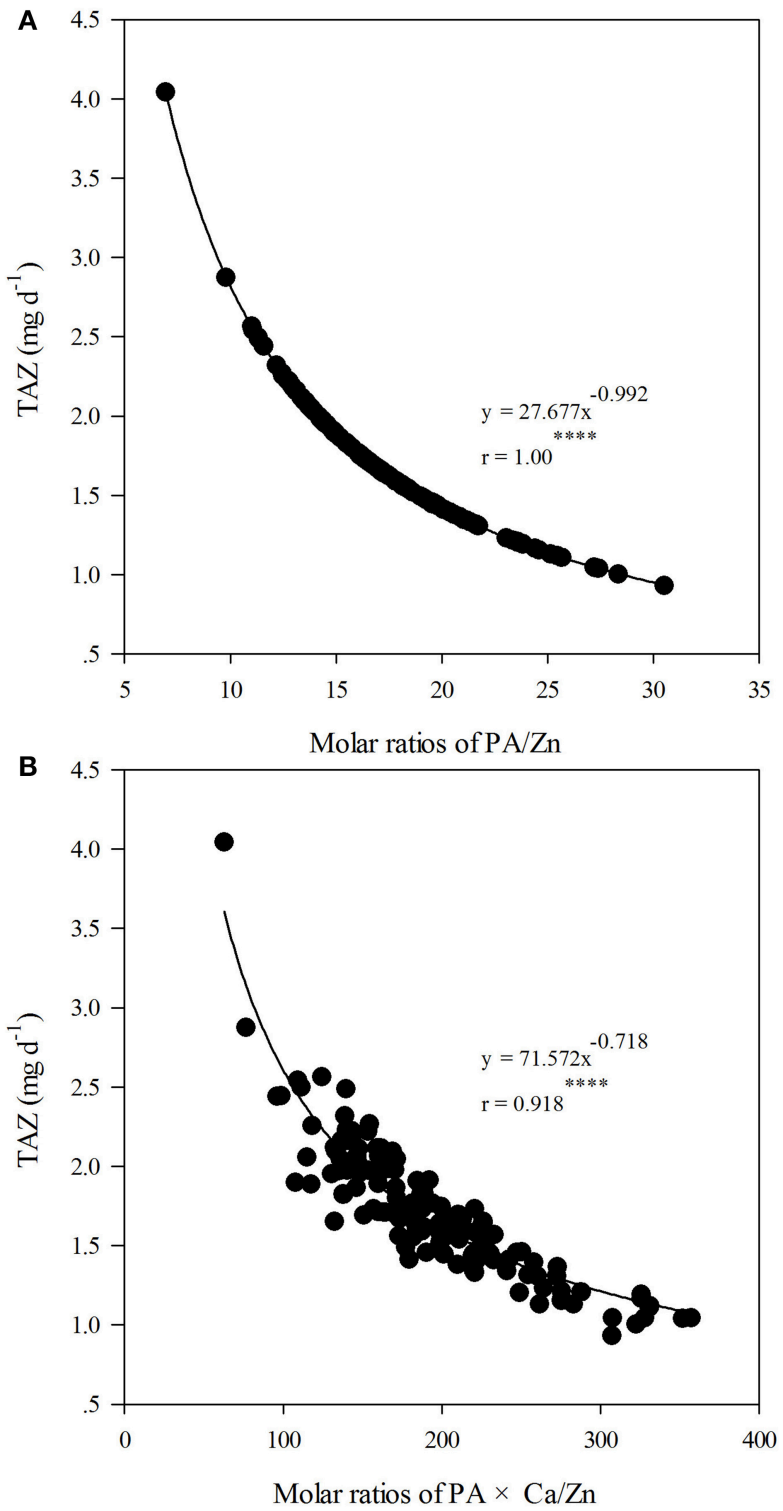
Relationships between total daily absorbed Zn (TAZ, mg d^−1^) and molar ratios of phytic acid (PA)/Zn **(A)**; and between TAZ and molar ratios of PA × calcium (Ca)/Zn **(B)** in whole flours across all treatments of nitrogen (N) application, cultivars and foliar spraying (*n* = 144). “****” indicates significant at *P* ≤ 0.0001.

Considering all 144 data points in this study, the molar ratios of PA/Zn and PA × Ca/Zn varied from 6.9 to 30.5 and from 62.7 to 357.2, respectively, and the TAZ ranged from 0.93 to 4.0 mg d^−1^. Figure 1 shows the relationship between TAZ and PA/Zn, which can be precisely described by a negative power function (R2 = 1.00^****^), while the relationship between TAZ and PA × Ca/Zn can be described by another negative power function (R2 = 0.85^****^). According to the fitted curves, the critical value of 15 for PA/Zn and 200 for PA × Ca/Zn corresponded with TAZ values of 1.9 and 1.6 mg d^−1^, respectively.

There were also significant and negative correlations between spike numbers and kernel numbers per spike, 1,000 kernel weights, phytate-P, total P, Ca concentrations, or molar ratios of PA × Ca/Zn; however, correlations between spike numbers and grain yields, Zn yields, C concentrations, or C/N ratios were all positive. Kernel numbers per spike were also negatively correlated with protein concentrations, but positively correlated with 1,000 kernel weights and Ca concentrations. One thousand kernel weights were also negatively correlated with C concentration, and positively correlated with phytate-P, total P, Ca concentrations, and molar ratios of PA/Zn and PA × Ca/Zn (Table 6).

Grain yields were positively correlated with Zn yields and C/N ratios, and Zn yields were negatively correlated with molar ratios of PA/Zn and PA × Ca/Zn. C concentrations were also negatively correlated with phytate-P concentrations, and concentrations of protein, Ca, and phytate-P were all negatively correlated with C/N ratios. In addition, positive correlations were observed among concentrations of Ca, phytate-P and total P, phytate-P/total P and molar ratios of PA/Zn and PA × Ca/Zn, except for a negative correlation between phytate-P/total P and total P (Table 6).

## Discussion

### Optimal fertilizer N application rate maintained high grain yields and enhanced grain Zn and protein concentrations as well as bioavailability

In 2015, a “Zero Increase Action Plan” was formally announced by the Ministry of Agriculture to reduce national fertilizer (e.g., N) use by 2020 (Liu et al., 2016); in practice, China is undergoing a transformation from the overuse of N to reasonable N input in wheat production (Chen et al., 2011, 2017), and our research showed that this transformation would have no negative effects on grain yield, Zn concentration, or bioavailability estimated using TAZ, PA/Zn, and PA × Ca/Zn (Tables 1, 2). Compared to adequate N supply (200 kg ha^−1^), excessive N application (275 kg ha^−1^) did not make an extra contribution to wheat growth, grain Zn concentration, Zn yield, bioavailability, or protein concentration, but resulted in a slight decline in grain yield, Zn concentration, and Zn yield at maturity (Tables 1, 2, 4). This is consistent with Xue et al. (2012), who found that further increasing N supply from the optimal value (198 kg ha^−1^ in the first year and 195 kg ha^−1^ in the second year) to excessive N supply (297 and 292.5 kg ha^−1^, respectively) caused non-significant increases in grain Zn concentration and content. However, N deficiency is still prevalent in many other developing countries, which may not only limit grain yield increase, but also lead to low grain Zn concentrations (Chen et al., 2017). In the current study, increasing N supply from the low level of 75 kg ha^−1^ to the optimal/adequate level of 200 kg ha^−1^ effectively improved grain Zn nutritional qualities. Therefore, rational N supply is necessary to maintain high grain yield for food quantity security, and it is also beneficial for the improvement of grain Zn nutrition for human dietary quality.

### Foliar Zn spraying is an effective way to improve grain Zn nutritional quality

Foliar Zn spraying with or without sucrose did not affect yield traits of wheat (Table 1). Similar results were previously reported (Cakmak et al., 2010a,b; Wang et al., 2012; Zhang et al., 2012b; Zou et al., 2012; Zhao et al., 2014), indicating that the grain yield was less dependent on exogenous foliar Zn and/or carbohydrate supply in most cases. There is one exception that foliar Zn spraying increased grain yield under drought conditions, even in a soil with high DTPA-Zn (Karim et al., 2012), suggesting that the exogenous Zn effectively supplemented the demand for Zn of wheat plants and reduced the drought-induced oxidative cell damage due to the improved antioxidative defense ability (Cakmak, 2000).

In agreement with the literature, foliar Zn supply alone significantly increased grain Zn accumulation (Cakmak et al., 2010a; Wang et al., 2012; Zhang et al., 2012b) as well as estimated Zn bioavailability in this study (Table 2). As reported previously, there was significantly positive correlation between grain Zn concentration and leaf concentration, indicating the importance of the pool of physiologically available Zn within vegetative tissues which are effectively utilized for grain Zn deposition after flowering (Haslett et al., 2001; Cakmak et al., 2010a; Kutman et al., 2010; Zou et al., 2012). Therefore, maintaining high amounts of Zn in leaves by foliar Zn spraying would undoubtedly contribute to the increase in grain Zn concentration as shown in the current study. According to reports from Graham et al. (2007) and Cakmak et al. (2010a), the current marked increase in grain Zn concentration (11.1 mg kg^−1^ in average, > the target of 10.0 mg kg^−1^) caused by foliar Zn application alone would have a measurable impact on improving dietary intake of Zn by human beings and human health. In addition to bran, the increase in wheat grain Zn concentration also occurs in endosperm, where Zn has higher bioavailability (Cakmak et al., 2010a). It is worth pointing out that foliar Zn spraying represents an effective way to grain Zn biofortification of wheat.

Remarkably, a synergistic “Zn + sucrose” foliar spray increased grain Zn concentration, content, and bioavailability more so than Zn alone. The relatively higher effectiveness of “Zn + sucrose” can be attributed to three aspects of sucrose: (1) a longer drying time of the Zn solution; (2) improved leaf cuticle penetration; and (3) improved translocation rates of Zn from the site of absorption to grains, as suggested by Zhao et al. (2014). Fernández et al. (2017) reviewed that specific additives can be used to lower the deliquescence relative humidity (DRH) of spray solutions to increase the rate of foliar absorption. In addition, swelling of the cuticle by absorption of substantial amounts of water may form “water-filled pores” (Schreiber, 2005) allowing the penetration of hydrophilic solutes across the cuticle (Fernández et al., 2017). Sucrose may have played such roles in lowering the DRH and facilitating swelling of the cuticle due to its strong ability to absorb and hold water. Our present study showed that foliar sucrose supply did not lead to significantly higher C concentrations and ratios of C/N, but led to slight declines and a significantly higher protein concentration (Table 4). In addition, no significant correlations were observed between grain Zn and C concentration, nor between TAZ and C or C/N, but grain Zn concentration was negatively correlated with C/N (Table 6). Therefore, we speculate that the foliar sucrose had not entered into the leaf cells or been translocated from leaves to grains, accompanying Zn, thus the distinguished effect of “Zn + sucrose” may be more dependent on aspects of (1) and (2), which need to be further studied.

### Cultivars showing higher grain yields and concentrations of antinutritional compounds had lower grain Zn nutritional quality

Many studies showed that there is a contradiction between grain yield and nutrition quality in crop breeding. For example, although the low phytate wheat lines have been used in breeding to improve grain Zn bioavailability, research along these lines has so far failed because of the associated 8–25% yield reduction (Guttieri et al., 2004, 2006). Some previous studies have also found that wheat grain Zn concentrations negatively correlated with grain yields (a “dilution” effect) or cultivar release years among diverse wheat cultivars and regions (Zhao et al., 2009; Velu et al., 2014). However, agronomic management approaches (e.g., fertilization) have not been given careful consideration in these studies (Chen et al., 2017). Many results presented in this study can confirm the above statement. Cultivars obviously differed in grain yields, yield components, grain Zn concentrations, bioavailability, and other nutritional traits (Tables 1, 2, 4). The grain Zn bioavailability of the most commonly used high-yielding “Jimai 22”—which exhibits the highest grain yields and concentrations of antinutritional compounds including phytate-P, total P, and Ca—was the lowest among the four wheat cultivars investigated. In contrast, “Jinan 17,” as a high-quality strong gluten wheat cultivar with relatively lower grain yields than “Jimai 22” and the lowest concentrations of phytate-P, total P, and Ca, exhibited the highest grain Zn bioavailability. Grain Zn concentrations were negatively correlated with grain yields, PA/Zn, and PA × Ca/Zn (Table 6). TAZ was negatively correlated with concentrations of phytate and total P, Ca, and molar ratios of PA/Zn and PA × Ca/Zn. Phytate and total P, phytate-P/total P, and Ca were all positively correlated with PA/Zn or PA × Ca/Zn. All of these results indicated that the higher the grain yields and concentrations of antinutritional compounds, the lower the grain Zn nutritional quality will be. It is worth mentioning that rational N application and foliar “Zn + sucrose” spray maintained high grain yield and simultaneously increased grain nutrition quality in relation to protein concentration, grain Zn concentration, and bioavailability, irrespective of the wheat cultivar. Foliar Zn supply decreased the concentration of total phosphorus, and there was a significant and positive correlation between grain Zn and protein concentrations.

### Integrated strategies for Zn biofortification

Wang et al. (2012) found Zn concentrations in grains of wheat varied from 18.79 to 23.11 mg kg^−1^ in the control treatment of soil containing 0.78 mg kg^−1^ DTPA-Zn without soil Zn application. Gomez-Coronado et al. (2017) reported grain Zn concentrations varied from 14.0 to 20.3 mg kg^−1^ when the DTPA-Zn was 0.28 mg kg^−1^ without soil Zn application, which is lower than 0.5 mg kg^−1^ DTPA-Zn as the widely recognized critical Zn level (Sims and Johnson, 1991). It is clear that the range of Zn concentrations between 34.9 and 47.7 mg kg^−1^ in the non-foliar Zn treated cultivars of this preset study (Table 3) is much higher than those obtained from Wang et al. (2012) and Gomez-Coronado et al. (2017), properly due to different wheat cultivars investigated and a high DTPA-Zn (1.5 mg kg^−1^) in combination with soil application of 30 kg ZnSO_4_·7H_2_O ha^−1^. In the location of Samsun with 1.59 mg kg^−1^ DTPA-Zn and Adana with 0.49 mg kg^−1^, at the rate of 50 kg ha^−1^ of ZnSO_4_·7H_2_O application to soil, Cakmak et al. (2010a) found grain Zn concentrations varied from 23 to 29 mg kg^−1^ and from 32 to 37 mg kg^−1^ in the controls without foliar Zn application, respectively, which indicated that a higher DTPA-Zn didn't necessarily lead to a higher grain Zn concentration. For example, the soil water content and precipitation are also considered as a major factor influencing grain Zn accumulation (Gomez-Coronado et al., 2016). In addition, a large genotypic variation in grain Zn concentrations was observed by Gomez-Coronado et al. (2016). The variation found in increases caused by foliar Zn application was also wide in different experiments. The current study showed a 27.1–38.1% increase in grain Zn concentrations treated by foliar Zn applications with or without sucrose. Lower increases were found by Cakmak et al. (2010a), of 14.9–35.0% in the location of Konya with 0.18 mg kg^−1^ DTPA-Zn, with foliar Zn applications alone at the soil application rate of 50 kg ZnSO_4_·7H_2_O ha^−1^. Higher increases were found by Cakmak et al. (2010a), of 44.8–106.9% in locations of Samsun and Adana, by Zhang et al. (2012b), of 39.5–72.9%, by Wang et al. (2012), of about 54.0%, or by Zou et al. (2012), of about 83.5%, with foliar Zn applications alone. Even much higher increases were reported by Gomez-Coronado et al. (2016), varying from the initial grain Zn concentrations of 12–20 mg kg^−1^ to 42–52 mg kg^−1^, as affected by foliar Zn supply alone. Therefore, different results were obtained from different experiments, it is complex to develop a most effective Zn biofortification strategy, all factors including cultivars, soil and other environmental conditions, and artificial managements (e.g., fertilization and foliar application times) should be considered and managed in whole and in a proper way.

Different organizations or scientists have set different Zn biofortification target levels in wheat grains to fulfill the Zn demand by human body. As far as we know, 38 mg kg^−1^ is set by the HarvestPlus program (http://www.harvestplus.org; Hao et al., 2015), 40 mg kg^−1^ is proposed by Allen et al. (2006) and Tang et al. (2008), and 45 mg kg^−1^ is determined by Ortiz-Monasterio et al. (2007) and Pfeiffer and McClafferty (2007), and 40–50 mg kg^−1^ is according to the FAO (Wang et al., 2012). In addition, the grain Zn concentration is not the only important factor, their bioavailability is also crucial to improve the daily Zn intake by human body. The molar ratios of PA/Zn and PA × Ca/Zn have been suggested as indicators of Zn bioavailability (Morris and Ellis, 1982; Ellis et al., 1987; Ryan et al., 2008). A molar ratio of PA/Zn below the critical ratio of 15 (World Health Organization, 1996; Gargari et al., 2007) and a molar ratio of PA × Ca/Zn below the critical ratio of 200 (Ellis et al., 1987) indicate a good Zn bioavailability. Molar ratios of PA/Zn of most cereal products varied from 25 to 34, indicating a low Zn bioavailability (Welch and Graham, 2002). It seems that grain Zn concentrations of “Jinan 17,” “Jimai 20,” and “Luyuan 502” (38.6–47.7 mg kg^−1^) in the controls of this study without foliar Zn and foliar “Zn + sucrose” supply are adequate (Table 3), especially according to 38 mg kg^−1^ set by the HarvestPlus program and 40 mg kg^−1^ by Allen et al. (2006) and Tang et al. (2008). However, their grain Zn bioavailability is still low, especially according to values of the most widely used indicator PA/Zn (15.3–27.7) (Table 3). The current research confirms that foliar Zn spraying alone or with sucrose is necessary to enhance both Zn concentrations and especially Zn bioavailability in combination with rational N supply.

In this study, the strategy of foliar Zn spraying was more effective than soil fertilizer N application to biofortify wheat with Zn. Compared with no Zn application, Zn spraying (with or without sucrose) increased grain Zn concentrations by 11.1–15.6 mg kg^−1^ (27.1–38.1%), and increased grain Zn bioavailability, estimated using TAZ, PA/Zn, and PA × Ca/Zn, by 0.4–0.6 mg d^−1^ (28.6–42.9%), 23.1–27.4%, and 24.0–28.0%, respectively. Increases caused by N application were only 2.1–4.7 mg kg^−1^ (4.4–9.9%), 0.2 mg d^−1^ (12.5%), 9.7–10.3%, and 6.9–7.4%, respectively. Among different wheat cultivars, grain Zn concentrations only varied from 47.0–52.9 mg kg^−1^, while TAZ, PA/Zn, and PA × Ca/Zn dramatically varied from 1.4 to 2.0 mg d^−1^, 14.7–20.7, and 140.1–249.1, respectively, suggesting that grain Zn bioavailability was more likely to be influenced by cultivar.

Here, we propose an integrated strategy to improve/maximize the grain Zn nutritional quality while ensuring high yields and protecting the environment. At least three factors above should be managed in coordination in wheat production: (i) adoption of biofortified cultivars with low phytate, high grain Zn concentration and/or bioavailability, high yield, and high resistance to stresses; (ii) optimization of a N fertilizer application amount that ensures higher grain yield, better Zn nutritional quality and lower N loss; and (iii) creation of an adequate available Zn pool in wheat shoots for its re-translocation to grains by efficient and/or economic foliar Zn application after anthesis in combination with soil Zn application according to the initial soil DTPA-Zn status before sowing. Under such a scenario, the target for biofortification will be rapidly achieved by combining agronomic and genetic strategies. The target value for wheat grain Zn biofortification is set to be 38–50 mg kg^−1^ (Allen et al., 2006; Ortiz-Monasterio et al., 2007; Pfeiffer and McClafferty, 2007; Tang et al., 2008; Wang et al., 2012; Hao et al., 2015). In the present study, the target value was completely achieved by rational N combined with foliar “Zn + sucrose” application for “Jinan 17” and “Luyuan 502.”. In addition, the molar ratios of PA/Zn and PA × Ca/Zn of “Jinan 17” or “Luyuan 502” were much <15 and 200, respectively, and the TAZ of “Jinan 17” and “Luyuan 502” was maximized to be 2.2 and 2.1 mg d^−1^, respectively, indicating higher grain Zn bioavailability. Recently, TAZ was used to calculate the health impact (disability-adjusted life years (DALYs) saved) of biofortified wheat flour and its reduction of the current health burden by Liu et al. (2017), who found that a greater available Zn intake (0.24–0.7 mg d^−1^) in whole flours reduced the health burden (DALYs) caused by Zn deficiency by 6.58–18.21%. Therefore, optimal N and foliar Zn management combined with suitable cultivars will eventually and substantially increase the Zn concentration and bioavailability, and the health impact of whole wheat flours, and contribute to mitigate the health burden of Zn deficiency among infants and children.

## Conclusion

In the current study, enhanced N application rate increased both the Zn concentration and bioavailability in whole wheat flours; excessive fertilizer N input did not improve either of them further. The strategy of foliar Zn spraying was more effective than soil fertilizer N application to biofortify wheat with Zn, especially for foliar Zn supply combined with sucrose. Compared to small variations in grain Zn concentrations, the grain Zn bioavailability was more sensitive to the selection of cultivar. Among different wheat cultivars, the higher the grain yields and concentrations of antinutritional compounds (e.g., PA), the lower the grain Zn nutritional quality will be. All of the results indicated that Zn biofortification of wheat through optimal soil N and foliar Zn management combined with suitable cultivars can maintain high grain yield with lower N input and simultaneously increase the Zn concentration, bioavailability, and thus the health impact of whole flours.

## Author contributions

HX and Yanfang Xue: conceived and designed the experiments; WK, Yanhui Xue, YT, JL, DoL, and PM: performed the experiments; HX, Yanfang Xue, and DuL: analyzed the data and wrote the paper.

## Conflict of interest statement

The authors declare that the research was conducted in the absence of any commercial or financial relationships that could be construed as a potential conflict of interest.

## Footnotes

^1^Available online at: http://www.fao.org/faostat/en/#data
